# A qualitative social network analysis of decision-making around child marriage in three villages in Bangladesh

**DOI:** 10.3389/fgwh.2026.1668789

**Published:** 2026-05-04

**Authors:** Anja Zinke-Allmang, Ruchira Tabassum Naved, Daniel J. Carter, Melissa Neuman, Leah Kenny, Raafat Hassan, Md. Ashraful Haque, Aklima Akter, Kaokaba Tun Nesa, Md. Masum, Nusaiba, Amiya Bhatia

**Affiliations:** 1 Department of Social Policy and Intervention, University of Oxford, Oxford, United Kingdom; 2 Faculty of Epidemiology and Public Health, London School of Hygiene and Tropical Medicine, London, United Kingdom; 3 Maternal and Child Health Division, icddr,b, Dhaka, Bangladesh; 4 Department of Anthropology, Shahjalal University of Science and Technology, Sylhet, Bangladesh

**Keywords:** adolescents, Bangladesh, child marriage, early marriage, social network analysis, social norms

## Abstract

**Background:**

In Bangladesh, the prevalence of child marriage remains high. Parents are known to be key decision-makers for their daughter's marriage, however, little is known about wider social network support for girl's early marriage. This study examines the key actors and dynamics in social networks, often across generations, that support early marriage decisions and explores the reasons, experiences, and perceptions of benefits for girl's early marriage.

**Methods:**

This qualitative social network analysis (SNA) interviewed 6 adolescent girls married before 18 years of age and members of their familial and social networks (*n* = 20) in three villages in Bangladesh. Young women and network members participated in semi-structured in-depth interviews and identified and ranked network members involved in marriage decisions. A thematic analysis was conducted and social network maps were created with participants.

**Results:**

Parents were central in the decision-making of girls' marriage. Fathers were final decision-makers and mothers were important in the coordination of marriage. The age of a girl was not a central factor in marriage decisions, rather readiness for marriage depended on perceptions of physical growth, maturity, demeanour, and beauty. Timing of girls' marriage was also based on household economic conditions and averting potential losses of honour, which were persistent themes across participants and generations.

**Conclusions:**

This study found that marriage decisions for adolescent girls were made by girls' immediate social and familial networks. Programming and policy changes are needed to create community- and network-led change to shift perceptions of girls' readiness for marriage before 18 years.

## 1 Introduction

South Asia accounts for nearly half of the global prevalence of child marriage (1) and Bangladesh has the highest national prevalence: in 2019, 51% of women aged 20–24 years, were married before 18 years (2–4). The short- and long-term effects of early and child marriage on girls’ health and wellbeing are well-documented and include increased risk of birth complications, poorer mental health, lower educational attainment, and increased risk of intimate partner violence (5–10). Although, the national prevalence of child marriage in Bangladesh has been slowly decreasing, trends vary geographically (3, 11–13).

In Bangladesh, the Child Marriage Restraint Act (CMRA) (2017) and National Plan of Action to End Child Marriage (NPA-ECM) (2018) have set the minimum age of marriage for girls as 18 years. Current research has demonstrated how community poverty, limited educational opportunities for women, and lower dowry for younger girls contribute to child marriage in Bangladesh (12–15). Early marriage is further driven by gender inequities and social norms that control girls’ sexuality, mobility, interaction with male peers, and attempts to preserve honour and family reputation may explain why reductions in child marriage have stagnated despite laws prohibiting it (14, 16–18). Norms contributing to child marriage are held and sustained by members of social networks and shape the perceived ideal age of marriage, particularly among social networks which have fewer non-familial members to change norms underpinning child marriage (19, 20). Family members that take part in and make decisions about girls' marriage do so within normative and material contexts that shape and inform choices to marry daughters at early ages, including the influence of family and community members. In Bangladesh and other contexts, strong norms surrounding child marriage limit alternative options for girls outside of early marriage and is often framed as a positive option for the security and benefit for the girl and her family (19, 21, 22).

While studies across contexts, including Bangladesh, have explored parents' roles as decision-makers around child marriage and the influence of social norms on early marriage decisions (13–16, 23–26), few studies have explored the wider social networks supporting and enabling early and child marriage in Bangladesh, including intergenerationally. This qualitative social network analysis (SNA) study aims to identify the key actors and dynamics within girls' social networks (27–29) that support early marriage and explore the experiences and processes of girl's early marriage in three villages located in sub-districts with divergent contextual trends in child marriage in Bangladesh.

## 2 Materials & methods

### 2.1 Study design

We conducted a qualitative SNA that was part of a larger study to explore changes in child marriage, including examining social norms and processes surrounding of child marriage, in three areas of Bangladesh. We adapted Shell-Duncan's qualitative egocentric SNA methods (30) to examine the role of adolescent girls and their networks in decision-making for early marriage. Egocentric SNA centres the perspective of one individual, in this study adolescent girls (“ego”), and their self-identified key social connections (“alters”) (28). Participants identified alters as decision-makers, individuals who are directly involved with decisions for important matters for girls in their families, and influencers, individuals that are consulted or discussed important matters for girls with decision-makers.

### 2.2 Study sites and participants

The minimum age of marriage for girls in Bangladesh is 18 years, set by the CMRA in 2017 and enacted through the NPA-ECM in 2018. Study sites were selected from icddr,b's Health and Demographic Surveillance Sites (HDSS), which have collected birth and marriage registration data since 1966 (Matlab), 1999 (Chakaria), and 2016 (Baliakandi). These three sites were chosen to represent different patterns of child marriage trends in the period between 2018 and 2022: where child marriage had increased (Matlab), decreased (Baliakandi), and remained the same (Chakaria). Two villages within each site were selected with similar child marriage trends to the sub-district. We selected 6 married girls aged 15–18 years, 2 girls per study site, to participate. Adolescent girls who had completed in-depth interviews from the larger study were randomly selected to participate in this study.

### 2.3 Data collection procedures

This SNA had two phases: 1) semi-structured interviews with six adolescent girls (“egos”) and 2) semi-structured interviews with up to the four most trusted network members to each ego (“alters”). All interviews consisted of two components: first, name generator questions to create a list and map of trusted people in participant's networks (Supplementary Document A) and secondly, questions to explore and describe key decision-makers, influencers, events surrounding participant's marriage, with alters additionally asked to reflect on processes of marriage in their community. Interviewers created and discussed hand-drawn network maps with participants as part of the interview.

Interviews were conducted by gender-matched interviewers in Bangla in 2023. Interviewers were trained on child marriage, rights, gender norms, qualitative and SNA research methods, research ethics, and study tools. Participants provided informed consent prior to interview and egos provided permission and contact information for their top five most trusted alters, of which up to four were interviewed. All participants were given information about local services relevant to women's health and child marriage. Data collectors met daily to debrief from interviews and discuss emerging themes. Interviews were digitally recorded, transcribed, checked for quality, and translated to English. Social network information and participant sociodemographic data were entered into excel.

### 2.4 Data analysis

We digitised network maps using R and used these to identify patterns of the size, closeness, and overlapping individuals within and across networks. In parallel, we conducted a thematic analysis of the interview transcripts to explore the context surrounding marriage practices and timing, decision-makers and influencers of marriage across networks, and to cross-check network maps. An initial codebook was based on interview questionnaires and revised with emerging codes and themes using an open coding approach. Thematic findings were cross-checked with network maps.

### 2.5 Ethical approval

London School of Hygiene and Tropical Medicine (Ref 28420) and the Ethical Review Committee of icddr,b (Ref PR-23004) approved the study. Permission to conduct further analyses of these data was provided by the University of Oxford (Ref SPI_C1A_24_011).

## 3 Results

### 3.1 Participant demographics

Our sample included 6 adolescent girls (egos) and 20 alters (12 women, 8 men). At the time of interview, girls were 15–18 years old. Three girls were pregnant and 3 had children. Two girls' husbands lived abroad for work. Within and across networks and generations, women's ages of marriage were similar to egos' ages of marriage with an average of 16 years, whereas men were married on average at 23 (Supplementary Document B).

### 3.2 Social networks

**Figure 1:**
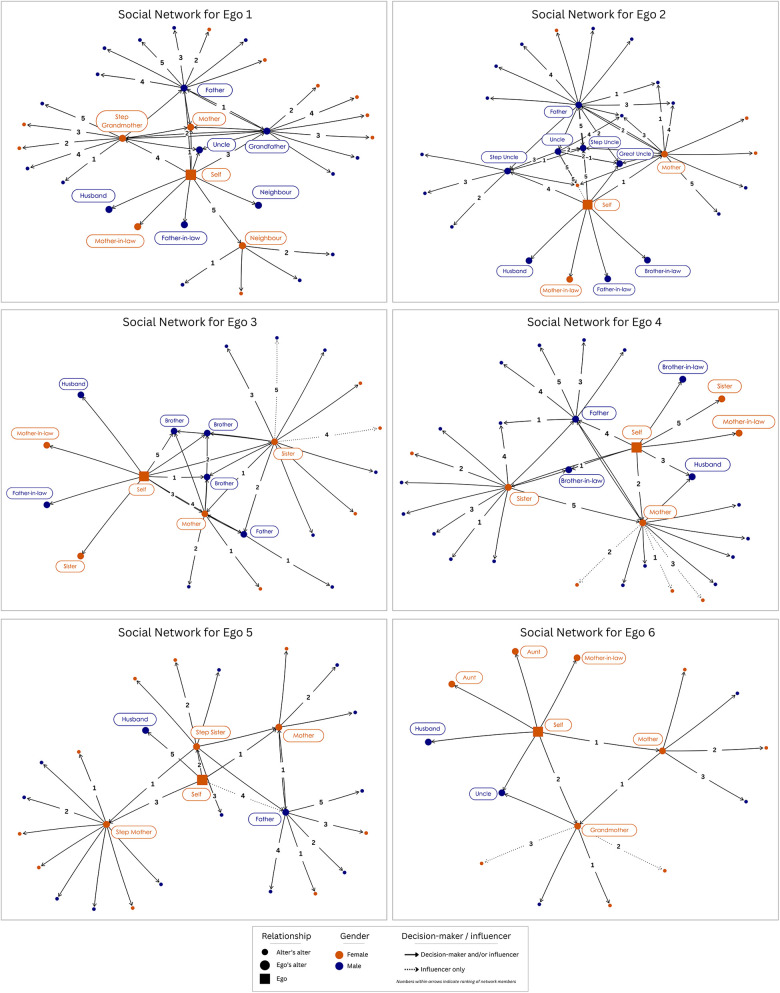
Egocentric social network maps of decision-makers and influencers of marriage among six adolescent girls.

Family members were the most trusted members of girls' social networks and included mothers, fathers, uncles, grandparents, neighbours, and siblings (Figure 1). Networks varied by size, closeness, and overlap of key individuals between ego and alter networks based on family context and structure. For example, Ego 2's network was extensive and close-knit, with many overlaps between alters suggesting a close network of decision-making within the family. In contrast, Ego 6's network was small and widely dispersed with few overlapping members. This network also had few male members following ego's parent's marital dissolution. Across all networks, parents were listed as decision-makers in every network and were the most important network members for adolescent girls. Across generations, alters described similar trusted relationships as egos, in which mothers and fathers were the most important (Supplementary Document C). Across all networks, members were mainly parents, uncles, and grandparents, indicating that parents' familial networks were the main influence for important decisions for girls in their families. Discussing or seeking advice for an upcoming marriage outside of the family was uncommon and none of the girls described friends as key supports, confidants, or influencers in their marriage.

### 3.3 Dynamics of decision-making within social networks

Parents were the most important decision-makers for a girl's marriage. All girls listed at least one parent whose decision on timing of marriage and choice of groom was ultimately followed: “*I was thinking, whatever my parents would decide, I will do that.*” (Ego 1) This was also true across generations, with parents commonly included as a key decision-maker or influencer in alter's networks. Although some girls described not agreeing with their parents' decision to be married, they felt that they needed to agree to the match because of familial expectations: “*I agreed [to marry] because of my family. Otherwise not*.” (Ego 3) While parents had the most prominent decision-making roles on marriage, participants also described a variety of roles for other family members (e.g., uncles, grandparents), such as visiting family and future in-laws, in marriage rituals, or in matchmaking.

While fathers were essential in finalising matches for marriage, they were often first approached by mothers or other family members who suggested the match. Most girls perceived their fathers as either supportive or not having strong opinions about the match: “*And whatever my mother (ammu) said my father (abbu) also agreed with that. He doesn’t talk much*.” (Ego 3) Most alters also saw fathers as decision-makers for girls' marriage in their families: “*A girl's father is the most important [person for decisions] naturally.*” (Grandfather of Ego 1) One girl's father suggested that she should follow her mother's suggestions for marriage: “*[My father said] keep calm, that means marry the one that your mom likes*.” (Ego 5) Fathers were perceived by girls to have spent minimal time on visiting natal family members to organise the wedding or the match, which often fell to other members such as mothers: “*Interviewer: What role did [your father] play to make the decision of your marriage? Participant: I didn’t see anything*.” (Ego 4) In Ego 6's family, where her father was not present, others such as a mother or grandmother took the role of finalising the match.

Mothers emerged across most networks as confidants and the person girls relied on the most. Often this support continued after they moved in with their marital families. Although mothers were often not the final decision-maker or matchmaker, they played a key role in persuading, conversing with, and bringing together family members to support the marriage across generations: “*My mother fixed the marriage and my father married me off*.” (Mother of Ego 3) All interviewed mothers identified themselves as important decision-makers for girls' marriage in their families. Most fathers also described mothers as closest to girls in their families, in charge of household matters, and involved in marriage processes, while fathers were the final decision-makers: “*This decision is taken firstly by mother. Mothers understand the intention of their daughters the best. The father does not understand the girl very well*.” (Father of Ego 1)

None of the six adolescent girls interviewed were actively involved in the choice of who or when they would marry: “*No one informed me [of the proposal]. I just heard. My mother told me […] after [my father and sisters]*.” (Ego 4) None of the adolescent girls in this study expressed wanting to be married and some described needing to be convinced to marry by family members: “*I agreed [to be married] because everyone told me to do. I did not agree before that.*” (Ego 2) However, some egos listed themselves as a decision-maker for their own marriage since they had been asked for their opinion or to agree with the family's decision on marriage. Some girls described wanting to pursue further education or find a job but felt that they needed to follow their parents' decision on marriage even if they did not agree: “*I won’t marry now, I will study. I will do a job after studying, a good job.*” (Ego 4) One girl described that she married early because her husband had blackmailed her family: “*That's why I married him, out of fear. He [said he] will upload [photos of us] on Facebook. So, I was scared. My mother teamed up with him. The entire family joined with him and I was alone.*” (Ego 5)

### 3.4 Decisions on the timing of girls' marriage

Decision-makers considered and were influenced by a variety of factors when considering the timing of girls' marriages, which were rarely based on age and rather stemmed from family's perceptions of girls' readiness for marriage. Markers of readiness for marriage include being out of school, physical maturity to carry a child and in appearance, and ability to manage a household. Network members also described poverty intersecting with readiness for marriage: “*In our area it's mostly seen that girls get married before 18 years as most of them are poor*.” (Uncle of Ego 2) Girls were aware of financial stress in their families, which convinced one girl to agree to her arranged marriage: “*We are also poor. I am poor, right?* *[...]* *I was thinking of [mother and grandmother's] decision [to marry me] and the question of money*.” (Ego 6) Other participants highlighted the links between honour, poverty, and impacts on girls' opportunities: “*Those who are poor, they marry off their girls due to the fear of losing honour. And those who have money, they let their daughters study and they think to let their daughters do jobs.*” (Neighbour to Ego 1)

Decision-makers also considered social pressures in addition to readiness for marriage when deciding on timing of girls’ marriage. Parents were concerned about the impact of gossip by community members on perceptions of girls' honour which would influence her marriageability: “*It's like whatever you say [people] will reply that your daughter has escaped. A little misunderstanding, maybe a quarrel with someone in the neighbourhood brings out the same words. [...] Parents give marriages out of this fear*.” (Father of Ego 1) Several other network members described early marriage arrangements to avert a potential loss of honour whereby a girl chooses to start a relationship or marry outside of the family's arrangements: “*There is no relevance with the age of the girls. They have to take care of their respect. If any girl elopes, then people will talk about it negatively. Then their respect will be harmed.*” (Grandfather of Ego 1)

Parent's concerns about love marriages and relationships impacting honour were amplified by increased access to mobile phones: “*Now girls are not taking husbands according to their parents’ choice. After the invention of mobile, they secretly make love affairs. [...] Now a person runs away with the person they like.*” (Stepmother of Ego 5) Parents and network members were concerned that young people use mobile phones to meet secretly: “*In our village, we see that the boys and girls use mobile phones and do fishy things—[having a] relationship is one of the things*.” (Father of Ego 4)

While age was not described as a central indicator of readiness to marry, adolescent girls and their networks referred to the legal minimum age of marriage when describing marriage practices. Some participants stated that early marriage may not be perceived as favourable, affecting marriage celebrations: “*My sister's (apu) marriage was arranged in a grand manner, whereas my marriage ceremony was not as extravagant, because I was underage. [I was] fifteen*.” (Ego 3) Network members were aware of the legal minimum age of marriage and described that underage marriage was common: “*[Girls] have to marry at least at the age of eighteen and otherwise it is not good as per the Government. [But] they are marrying at the age of fourteen to fifteen*.” (Mother of Ego 5) While several participants speculated that age of marriage would increase in the future because of the legal minimum age, one participant described workarounds to the law, such as giving bribes to officials and organising separate marriage celebrations later: “*They say that police would come if [young girls] get married at home. How can they do it at court when there is strict law? They do it taking bribe. Later, they arrange wedding ceremony at home but are married off at court*.” (Mother of Ego 3)

## 4 Discussion

This study draws on qualitative social network interviews with six adolescent girls married before the age of 18 and interviews with 20 important people in their social networks. We find that marriage decisions were kept within close family structures, evident from overlapping familial network maps between egos and alters. Similar to other literature on child marriage, we found that adolescent girls did not make decisions around their own marriages and rarely connected with their families and wider social networks for advice leading up to their marriages (14, 16, 31). Elder family members, particularly parents, have strong roles in influencing and deciding marriage timing and choice of groom for girls and young women in Bangladesh, which was based on markers of readiness than age (32, 33). We also found that marriage practices, perceptions of readiness for marriage, and key social network members and marriage decision makers were consistent across generations in families where adolescent girls had been married before the age of 18 years.

Our study elaborates on the distinct roles of fathers and mothers in girls' marriage arrangements. Consistent with other literature (33, 34), both egos and alters recognised fathers as important figures in the final decision about marriage for daughters and mothers were important figures as confidants and in persuading, conversing with, and bringing together key family members to support the marriage. Our findings highlight the importance of mothers as decision-makers in their daughters' marriages in a context where most literature shows that women's decision-making power is affected by norms that limit women's mobility and empowerment (35–37). We found that, while fathers are important final decision-makers, some girls perceived their fathers to have spent minimal time in finding matches or coordinating other family members to discuss or accept a girl's marriage, which was often done mothers or other family members. This discordance highlights that steps leading up to a final marriage decision involves many other influential network members and private decisions and discussions about marriage that do not involve adolescent girls. The importance of parents was also evident across generations, where social network maps revealed similar patterns of mothers and fathers as key decision-makers and influencers for marriage.

Despite the CMRA in 2017 and NPA-ECM in 2018 setting and prioritising minimum ages of marriage, we found dissonance between awareness of the legal minimum age of marriage for girls' and participants' experiences of marriage and ages of marriage in their communities. This gap in legal and actual age at marriage shows that laws do not directly translate into changes in practice (23, 33). Rather, we found that the timing of adolescent girls' marriage was closely linked to perceptions of readiness for marriage, as opposed to the focus on age seen in global health research and advocacy (14, 16, 24). When age was discussed by participants, it was in the context of physical and emotional maturity to bear a child, run a home, and being out of school. Especially among households in poverty, child marriage is perceived as a solution to reduce scarcity and financial stress in families, to avoid paying higher dowry prices at older ages, and an opportunity for economic security for their daughters (14, 15, 33, 38). However, child marriage is also underpinned by a constellation of gender norms that tie girls' sexuality to family honour and controls girl's mobility and engagement with boys and men (16). We found that parents were concerned about and would arrange marriages for their daughters at younger ages to avert a real or potential loss of honour through community gossip or a girl choosing to start a relationship (33, 38–40). As parents arrange marriages to protect girls' honour, girls' agency and involvement in decision-making in their own marriages are limited (14, 41). Despite aspiring to continue education or work rather than marry, many girls could not continue or return to their studies following their marriage. Allowing girls to remain in school and delaying their age of marriage has been linked to reducing various short- and long-term social and health consequences among girls (5–10), which requires governments to implement multisectoral approaches to prioritise girls’ human rights and support families to keep girls in school for longer, such as providing financial incentives and implementing community led programmes to change gender norms on child marriage (38). However, unlike in previous generations, parents and networks were now additionally concerned about girls' mobile phone use to connect with boys and men without their parents or family's awareness (15).

### 4.1 Strengths and limitations

This study is the first study to our knowledge to use qualitative SNA to explore child marriage decision-making in Bangladesh. A key strength of this study is that girls were interviewed directly about their experiences and their perceptions of decision-making alongside other family members to draw out tensions and areas of alignment. This study has several limitations. Qualitative SNA is a time-consuming and multi-step method, which can make it difficult to ensure interview questions are asked consistently to explore all influential network members. Since the focus of the interview was to visualise social networks, there was limited time to discuss interpersonal relationships in detail. This study was designed to be exploratory with a small sample size that provide illustrative processes and experiences of early marriage, thus findings are not designed to represent marriage experiences among adolescent girls in each site where findings may have been influenced by the small sample size. We did not conduct SNA among girls who were not married before the age of 18 years to explore differences in networks and marriage practices. Teasing out the difference between “decision-makers” and “influencers” was often unclear in the interview process.

### 4.2 Implications for programming, policy, and research

This study underscores the need for interventions on aiming to reduce child marriage to expand their focus to the social networks that decide on marriage for adolescent girls, rather than solely on adolescent girls who hold little decision-making power (42). Programmes seeking to change social norms surrounding child marriage should consider creating community-led and gender-transformative dialogues with key influencers to support new positive norms and shift perceptions of girls' readiness for marriage. Programme activities should specifically engage with families, boys, and men to facilitate gender equitable attitudes. Finally, programmes should also promote social protection measures to support households with economic instability and commit to women's economic empowerment and poverty alleviation. Future research should explore differences in decision-making processes and social networks among families which chose to marry daughters before and after age 18.

## 5 Conclusion

This study examined the social networks of six adolescent girls across three villages in Bangladesh and found that early marriage was driven by perceptions of readiness for marriage, household economic conditions, and parents' concerns about a loss of honour. Key decision-makers in adolescent girls' marriage include mothers, fathers, grandfathers, and uncles, with influence from siblings, aunts, and other close family members. Programming and policy changes are needed to create community- and network-led changes to shift perceptions of girls’ readiness for marriage before 18 years.

## Data Availability

The datasets presented in this article are not readily available because the datasets generated and/or analysed during the current study are not publicly available due to including sensitive and identifiable information. Additional data are available in appendices for this article. Requests to access the datasets should be directed to anja.zinke-allmang@spi.ox.ac.uk.
