# Supplementary material for: A qualitative social network analysis of decision-making around child marriage in three villages in Bangladesh
Source: Front Glob Womens Health. 2026 May 4;7:1668789. doi: 10.3389/fgwh.2026.1668789 (PMC13180723; doi:10.3389/fgwh.2026.1668789)

Supplementary Document C. *Social network maps of decision-makers and influencers of important decisions for girls for each Alter.*

Manuscript: *A qualitative social network analysis of decision-making around child marriage in three villages in Bangladesh*

Ego 1: Alters Social Network Maps

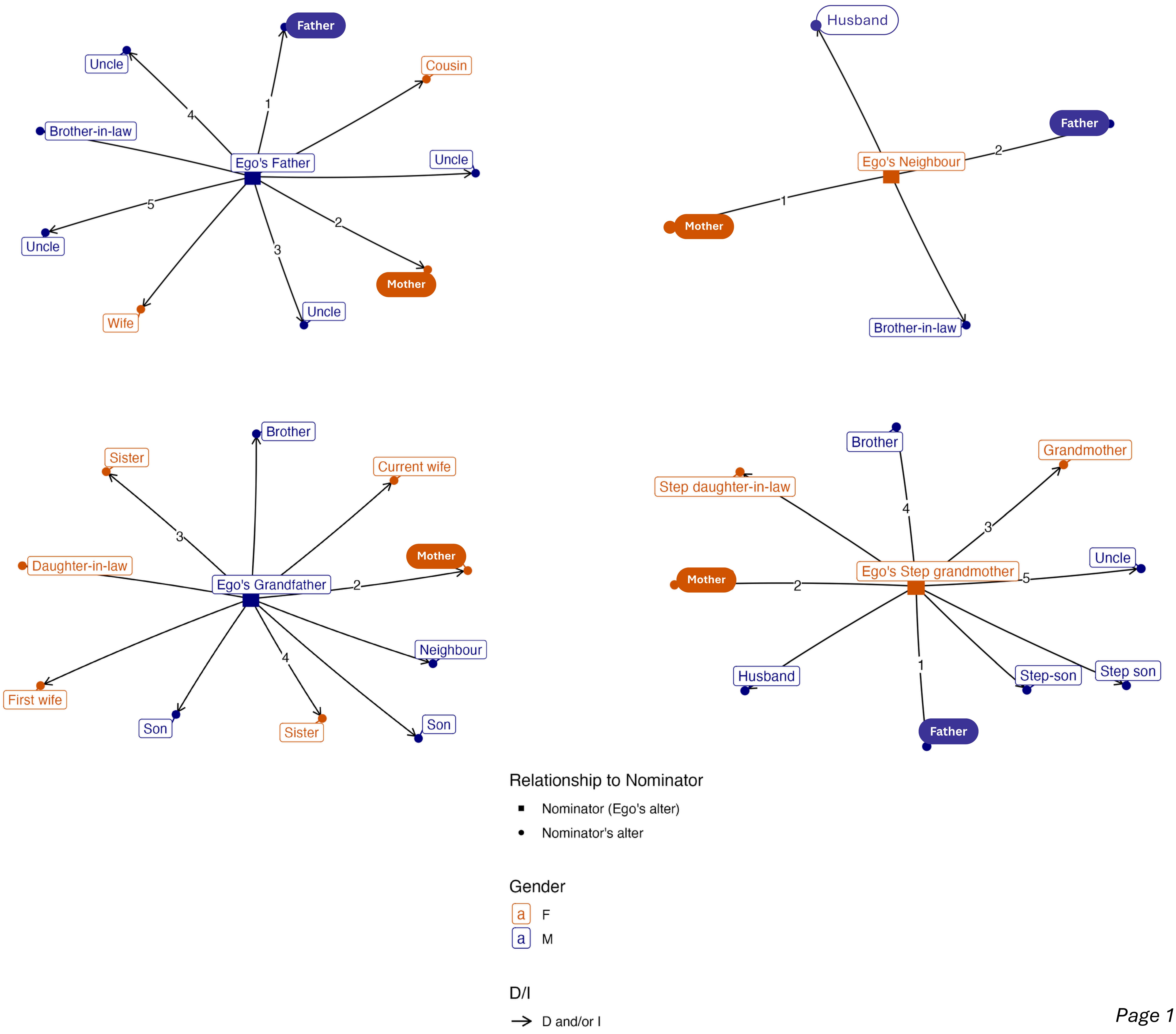

Supplementary Document C. *Social network maps of decision-makers and influencers of important decisions for girls for each Alter.*

Manuscript*: A qualitative social network analysis of decision-making around child marriage in three villages in Bangladesh*

Ego 2: Alters Social Network Maps

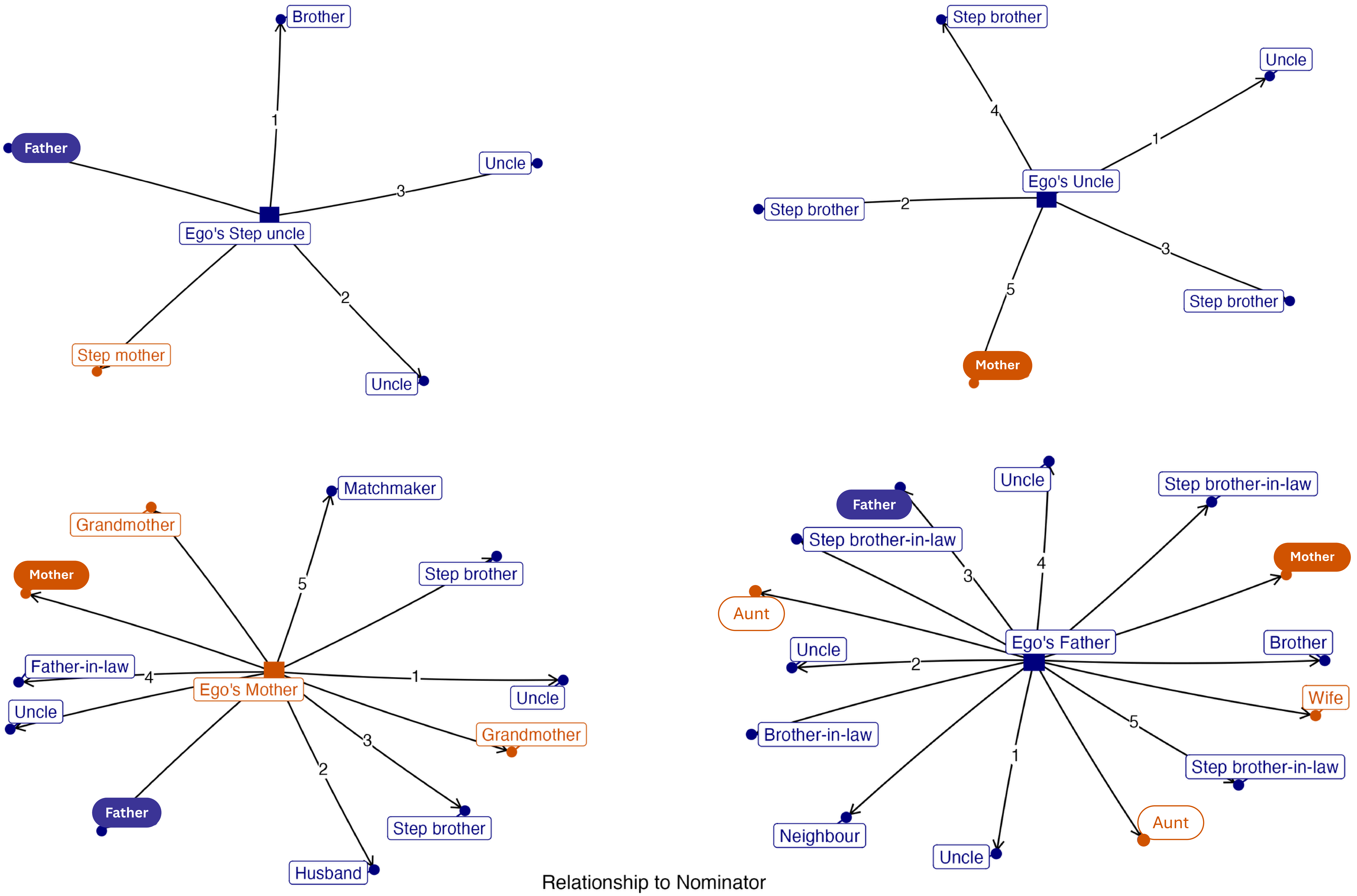

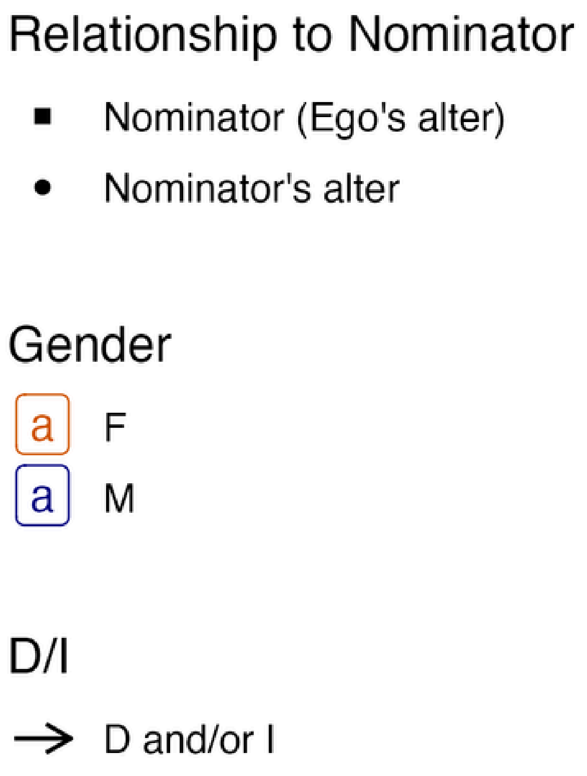

Supplementary Document C. *Social network maps of decision-makers and influencers of important decisions for girls for each Alter.*

Manuscript: *A qualitative social network analysis of decision-making around child marriage in three villages in Bangladesh*

Ego 3: Alters Social Network Maps

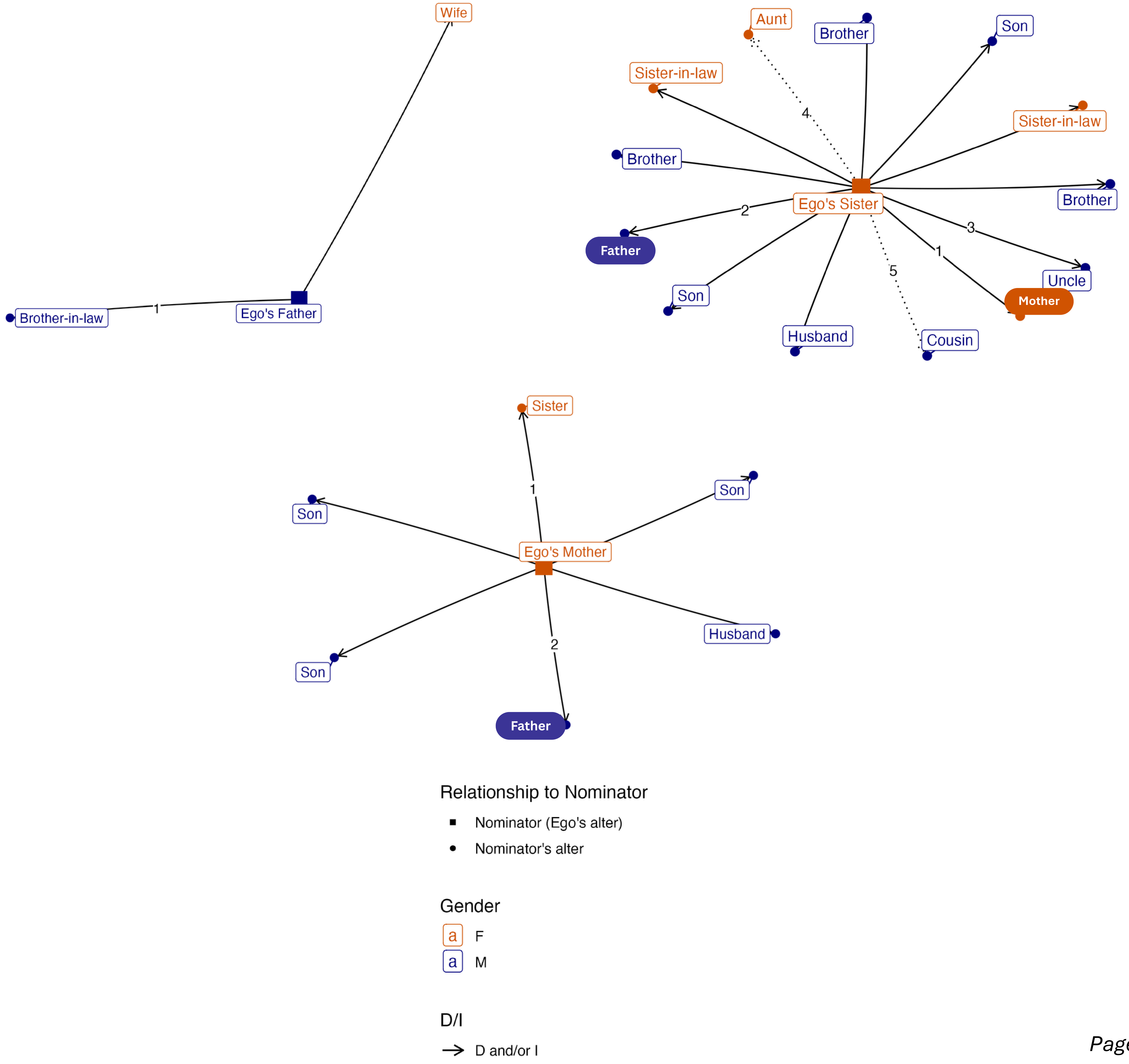

Supplementary Document C. *Social network maps of decision-makers and influencers of important decisions for girls for each Alter.*

Manuscript: *A qualitative social network analysis of decision-making around child marriage in three villages in Bangladesh*

Ego 4: Alters Social Network Maps

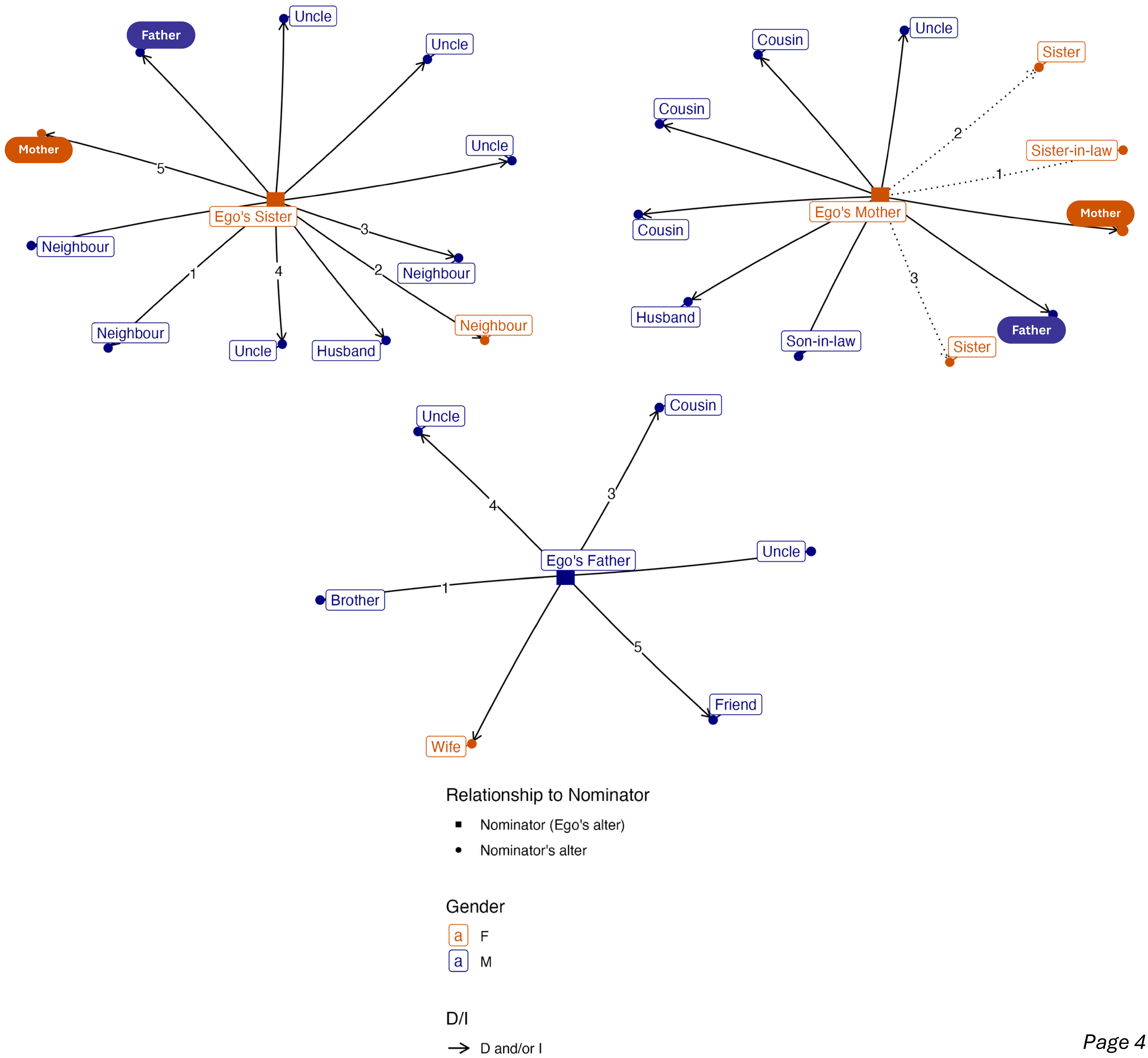

Supplementary Document C. *Social network maps of decision-makers and influencers of important decisions for girls for each Alter.*

Manuscript*: A qualitative social network analysis of decision-making around child marriage in three villages in Bangladesh*

Ego 5: Alters Social Network Maps

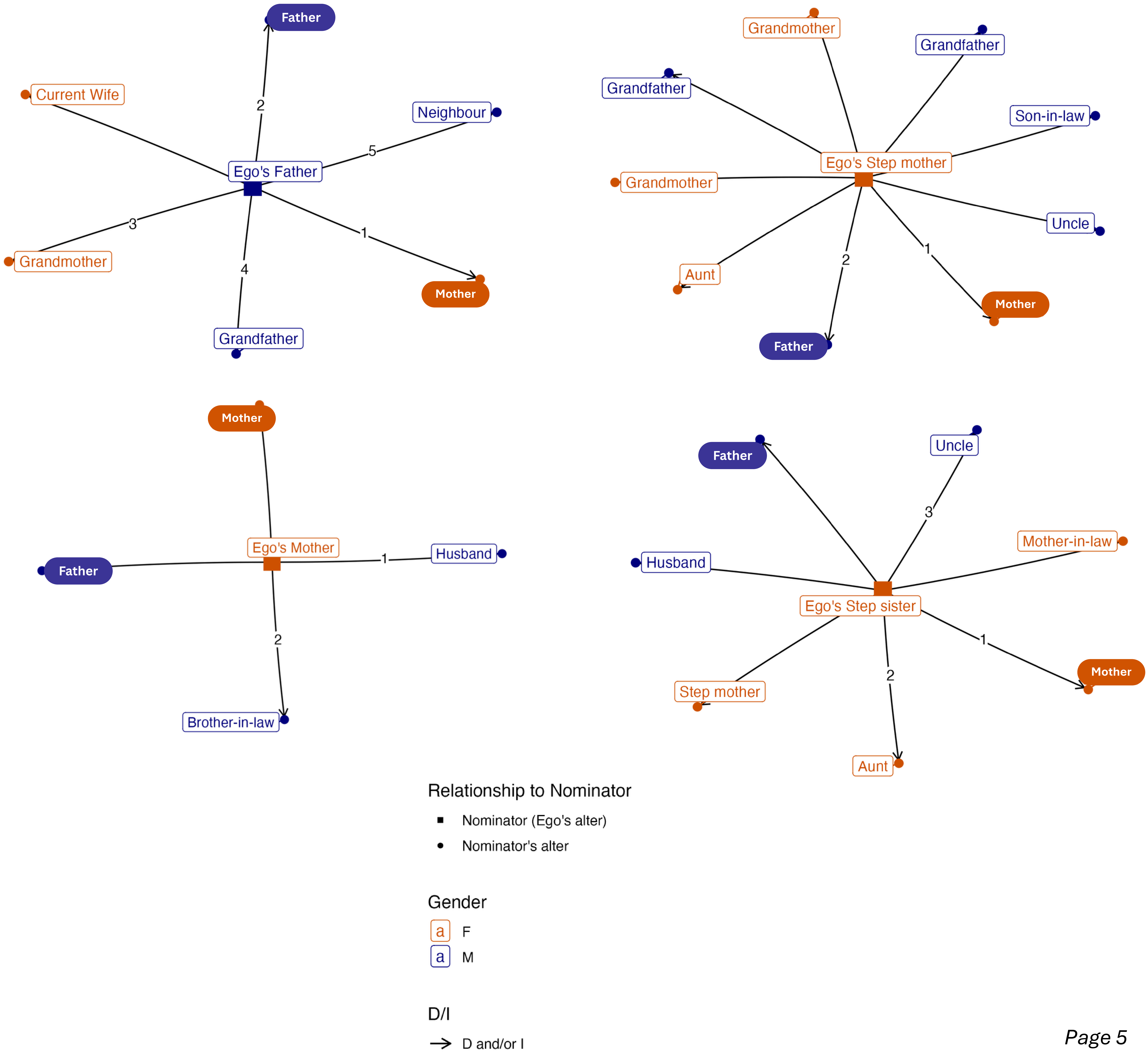

Supplementary Document C. *Social network maps of decision-makers and influencers of important decisions for girls for each Alter.*

Manuscript*: A qualitative social network analysis of decision-making around child marriage in three villages in Bangladesh*

Ego 6: Alters Social Network Maps

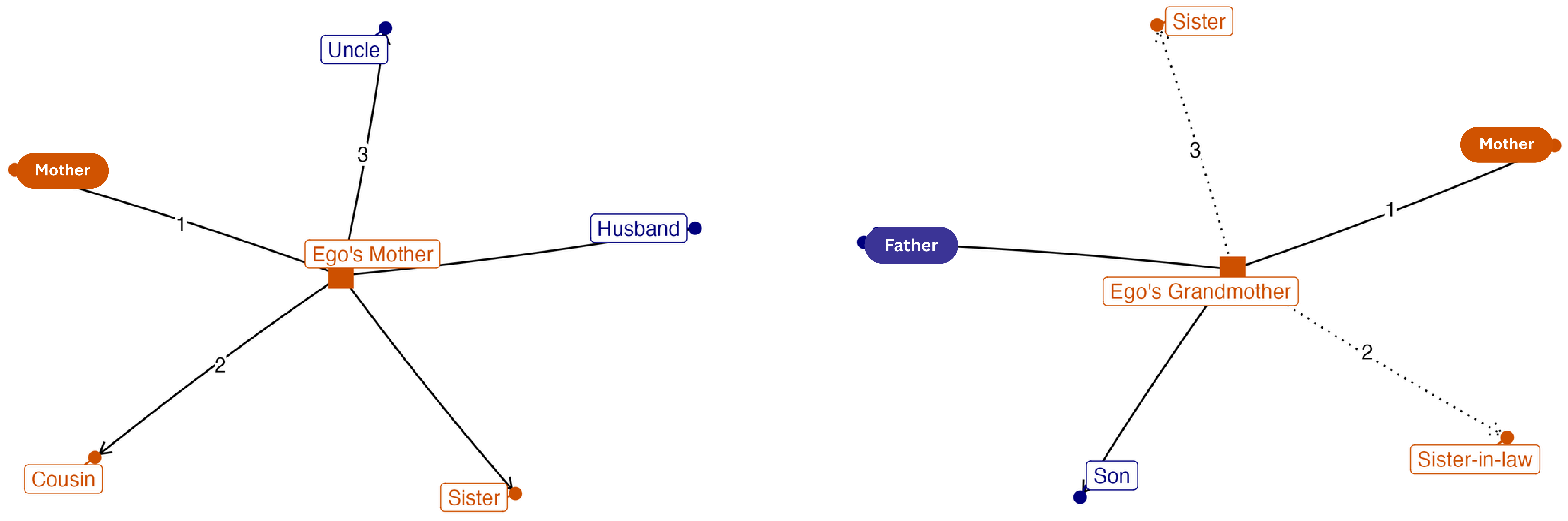

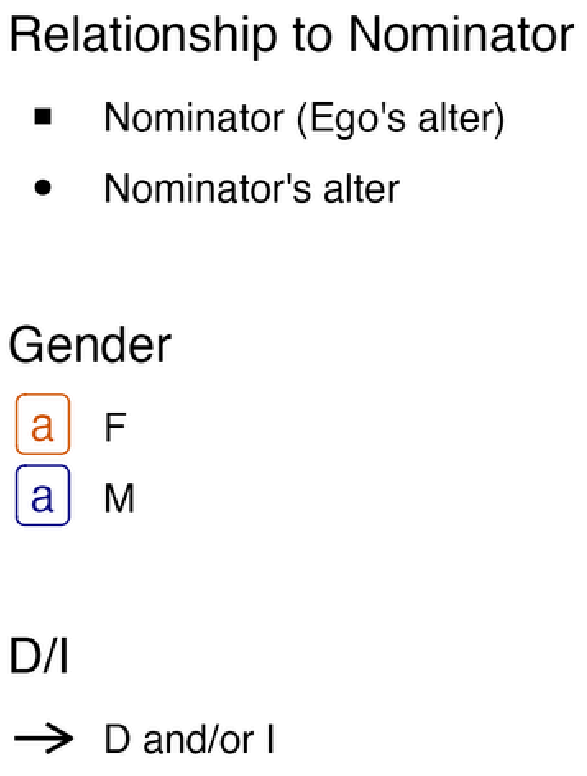

Supplement: Supplementary file 1 [file Datasheet1.pdf]
