# Supplementary material for: A qualitative social network analysis of decision-making around child marriage in three villages in Bangladesh
Source: Front Glob Womens Health. 2026 May 4;7:1668789. doi: 10.3389/fgwh.2026.1668789 (PMC13180723; doi:10.3389/fgwh.2026.1668789)
Supplement: Supplementary file 2 [file Datasheet2.pdf]

**Supplementary Document A. Listing questions to explore, list, and rank social networks among Egos and Alters**

The following questions are from semi-structured interviews with Egos and Alters to explore, list and rank their social networks. Interviewers drew maps with participants that included the people participants listed from these questions and maps were used as a tool to discuss and map social networks. Interviewers were trained in qualitative interviewing methods. Please see the full manuscript for more information.

*[This first set of questions were asked to Egos only] Now I want you to think about all the people in your life. People you talk to, know, spend time with, or speak to either often or occasionally.*

1. Who are the people that are most important to you?
2. If you feel worried or upset, who do you talk to?
3. Who do you talk to when making an important life decision?

*Thank you. I would now like to ask you about important decision makers in your family and draw a map of how you know these people.*

1. Who participates in important decisions around girls in your family?
2. Who decides if you should seek medical care when a girl in your family is sick?
3. Who is involved in the decisions around when a girl in your family gets married?

*Thank you. Thinking of the people who are in this map, I would like to talk to you about the people who are most important to you.*

1. Who did you trust the most to give you advice on your marriage? (name 1)
2. If this person was not available, who would you ask next? (name 2)
3. If this person was not available, who would you ask next? (name 3)
4. If this person was not available, who would you ask next? (name 4)
5. If this person was not available, who would you ask next? (name 5)

*Manuscript title: A qualitative social network analysis of decision-making around child marriage in three villages in Bangladesh.*

*Last updated: 01 May 2025.*
