# Supplementary material for: A qualitative social network analysis of decision-making around child marriage in three villages in Bangladesh
Source: Front Glob Womens Health. 2026 May 4;7:1668789. doi: 10.3389/fgwh.2026.1668789 (PMC13180723; doi:10.3389/fgwh.2026.1668789)
Supplement: Supplementary file 3 [file Datasheet3.pdf]

Supplemental document B. Sociodemographics of egos and the five most important people in her social network (alters)

Ego Attributes						Top 5 ranked trusted people *(ranked from most to least important)*					
Ego ID	*Age*	*Age at marriage*	*Area*	*Highest education level*	*Number of children*	*Relation to ego*	*Sex*	*Age*	*Age at marriage*	*Age at first child*	*Number of children*
Ego 1	18	17	Balikandi	Completed primary school	Pregnant at time of interview, 0 children.	Mother	F	Not interviewed			
						Father	M	41-45	21	22	2
						Grandfather (paternal)	M	61-65	17	19	4
						Step-grandmother (paternal)	F	36-40	19	20	4
						Neighbour	F	36-40	17	18	3
Ego 2	17	16	Balikandi	Completed some secondary school	Pregnant at time of interview, 0 children.	Mother	F	31-35	15	16	3
						Father	M	41-45	23	24	3
						Step-uncle (maternal)	M	Not interviewed			
						Step-uncle (maternal)	M	36-40	22	26	3
						Uncle (maternal)	M	31-35	24	25	2
Ego 3	16	15	Matlab	Completed some secondary school	0	Brother	M	Not interviewed			
						Sister	F	36-40	16	18	3
						Mother	F	61-65	15	16	6
						Father	M	71-75	32	35	6
						Brother	M	Not interviewed			
Ego 4	18	16	Matlab	Completed some secondary school	1	Sister	F	26-30	20	21	2
						Mother	F	56-60	18	19	3
						Husband	M	Not interviewed			
						Father	M	51-55	25	26	3
						Sister	F	Not interviewed			
Ego 5	15	14	Chakaria	Completed some primary school	Pregnant at time of interview	Mother	F	36-40	18	19	4
						Step-sister	F	20-25	16	17	2
						Step-mother	F	36-40	13	16	3
						Father	M	56-60	26	27	8

						Husband	M	Not interviewed			
Ego 6	18	17	Chakaria	Completed some primary school	0	Mother	F	31-35	16	17	1
						Grandmother (maternal)	F	56-60	18	19	5
Reasons participants were not interviewed include: working away from home at time of interview (for harvest, abroad), away from home travelling, sample size achieved of interviewing up to four alters per ego. Data was collected by qualitative social network interviews in 2023.											

Manuscript title: A qualitative social network analysis of decision-making around child marriage in three villages in Bangladesh.
Last updated. 01 May 2025.
